# Incidental neuroendocrine tumor of the appendiceal base less than20 mm in diameter: is appendectomy enough?

**DOI:** 10.11604/pamj.2015.22.102.6548

**Published:** 2015-10-06

**Authors:** Landolsi Sana, Mannai Saber

**Affiliations:** 1 Department of Surgery, Jendouba's Hospital, University of Medicine Tunis El Manar, Tunisia; 2 Department of Surgery, Mahmoud El Matri Hospital, University of Medicine Tunis El Manar, Tunisia

**Keywords:** Neuroendocrine tumors, appendix, treatment

## Abstract

The appendixis the second primary site for neuroendocrine tumors. The management of incidentelly discovered neuroendocrine tumor of the appendiceal base less than 20 mm in diameter is still controversal. The aim of this study was to discuss the management of such tumors. Three patients were operated on for acute appendicitis. Histopathologic examination of surgery specimens revealed neuroendocrine tumors of the appendiceal base less than 20 mm in diameter. Since no one presented with poor prognostic factors, no complementary right hemicolectomy was performed. No recurrence was observed. The existence of poorprognostic factors at histopathologic examination should indicate complementary right hemicolectomy for incidental neuroendocrine tumor of the appendiceal base less than 20 mm in diameter.

## Introduction

The appendix counts for the second primary site of neuroendocrine tumors occuring in 25 to 30% of the cases [1, 2]. These tumors are located in the base of the appendix in only 10% of the cases. They are discovered incidentally after appendectomy for acute appendicitis [3]. The management following appendectomy still controversial especially for tumours of less than 20 mm in diameter. The aim of this study was to discuss the management for incidentally histopathologic discovered neuroendocrine tumors of the appendiceal base with a diameter lessthan 20mm.

## Methods

The case series included three patients, two females and one male. The ages at diagnosis were 38 years, 42 years, and 45 years. All three patients were admitted for acute appendicitis. None suffered from the carcinoid syndrome. Appendectomy was performed laparoscopically in two cases and by elective laparotomy in one patient. Per operatively, The macroscopic aspects of the appendix were gangrenous in one patient and phlegmonous in two cases. Appendectomy was performed with uneventfull course. The histological analysis of the surgical specimens revealed the neuroendocrine tumors.

## Results

The three tumors were located at the base of the appendix, well-differentiated, without cellular necrosisnor vascular invasion. The tumor sizes were 5mm, 7mm, and 15 mm. Mitotic figures were 2 mitosis per 10 high-power fields in one patient, 3 in one case, and 4 in the other one. Proliferative activity Ki-67 was nil in two cases and 2% in one patient. Microscopic invasion was limited to the submucosa, the musculosa, and the subserosa in one case each. The surgical margins were negative for tumor cells. No mesoappendiceal involvement was found. Postoperative computerized tomography of the abdomen didn´t demonstrate metastases. The somatostatin receptor scintigraphy was normal. The secretion of 5-Hydroxy-Inndole-Acetic Acid measured after a 24-hour collection of urine was normal in all patients. No poor prognostic factors were found in our patients thus complementaryright hemicolectomy wasn´t carried out. No relapse was diagnosed after a follow up of 12 months.

## Discussion

As for our patients, appendectomy seems enough for neuroendocrine tumors of the appendiceal base less than 20 mm in diameter unless poor prognostic factors are present. Our results are in agreement with the litterature. The appendiceal neroendocrine tumors are discovered at a younger age than the othersites with a mean age of 42 years [4] as in our cases. It may be secondary to incidental diagnosis during appendectomy thatoccurs more frequently in younger patients. They are more frequent in women with a sex-ratio of 0,5 [5–7]. No specific clinical presentation is described [8]. Abdominal pain represents the most common complaint [5, 9]. Association with a carcinoid syndrome occurs in only 1% of cases [6]. Several factors are taken into account before deciding to perform complementary surgery. The most considered factor is the tumor size [2]. Since tumors greater than 20 mm in diameter increase the incidence of metastatic spread ranging from 20% to 85% [10, 11], they should be managed with a formal right hemicolectomy [2]. For those with a diameter less than 20 mm, appendectomy is enough unless an other poor prognostic factor is found. The indications for a complementary surgery include histological evidence of mesoappendiceal extension [12], tumor at the base of the appendix with positive margins or involvement of the caecum [13], high-grade malignant carcinoid tumor with a raised tumor prognostic index as measured by mitotic index and Ki-67 levels [12], lymph node involvement, and cellular pleomorphism with a high mitotic index [14]. The appendiceal neuroendocrine tumors have a good prognosis with 90,3% 5-year disease-specific survival [9].

## Conclusion

Appendectomy is enough for incidental neuroendocrine tumor of the appendiceal base less than 20 mm in diameter unless poor prognostic factors at histopathologic examination are present.
